# Corrosion behavior of hybrid boride–aluminide layers grown on selective laser melted Inconel 718

**DOI:** 10.1038/s41598-026-47359-z

**Published:** 2026-05-05

**Authors:** Barış Günay, Ali Günen, Ozkan Gokcekaya, Hasan Yildizhan, João Gomes

**Affiliations:** 1 Department of Metallurgy and Materials Engineering, Graduate School, Iskenderun Technical University, 31200 Iskenderun-Hatay, Turkey; 2 Faculty of Engineering and Natural Sciences, Department of Metallurgy and Materials Engineering, Iskenderun Technical University, 31200 Iskenderun-Hatay, Turkey; 3 Division of Materials and Manufacturing Science, Graduate School of Engineering, Osaka University, 2-1, Yamadaoka, Suita, Osaka 565-0871 Japan; 4 Energy Systems Engineering, Engineering Faculty, Adana Alparslan Türkeş Science and Technology University, 46278 Adana, Turkey; 5 Faculty of Engineering and Sustainable Development, University of Gävle, 801 76 Gävle, Sweden

**Keywords:** Additive manufacturing, Boronizing, Boroaluminizing, Aluminizing, Corrosion, Chemistry, Engineering, Materials science

## Abstract

The geometric freedom afforded by Selective Laser Melting (SLM) is increasingly driving the use of superalloy in advanced engineering applications. However, defects inherent to SLM, such as microsegregation, unmelted particles, and microporosity, weaken the alloy’s passive film continuity in chloride-containing environments, limiting its corrosion resistance. In this study, the microstructural evolution and electrochemical corrosion behavior of boron–aluminide coatings grown on SLM-Inconel 718 alloy through aluminizing (SSA), boronizing (SSB), simultaneous boroaluminizing (CBA), and sequential aluminizing-boronizing (SAB) and boronizing-aluminizing (SBA) processes were comparatively investigated. The coatings were applied at 980 °C using the pack boronizing/aluminizing method, and phase formations were analyzed by XRD, while layer morphology and elemental distributions were analyzed by SEM-EDS. Multiphase aluminide structures containing NiAl, Ni₃Al, FeAl₂, and Al₂O₃ were observed in the aluminizing process; Ni₂B, CrB, and FeB boride zones were observed in the boronizing process; and boride–aluminide mixture phases grown with varying degrees of homogeneity and continuity in the CBA, SAB and SBA processes. Layer continuity was most compact in the CBA and most disordered in the SAB. OCP and Tafel tests in 3.5% NaCl solution showed that corrosion behavior is directly related to layer integrity and porosity. According to the quantitative results, the corrosion current density was as follows: SBA < CBA < SSA < SSB < as-built IN718 < SAB. The highest performance was achieved in the SBA coating, with Icorr values ​​of 1.73 × 10⁻⁶ A/cm² and Rp of 3.06 × 10⁴ Ω being achieved thanks to the synergistic effect of the mechanical stability of the boride layer and the dense and adherent Al₂O₃ barrier formed by aluminizing processes.

## Introduction

Corrosion is an inherently destructive phenomenon in which metallic materials undergo electrochemical interactions with their environment, leading to progressive degradation of their properties and often resulting in a loss of functionality^1,2^. Except for noble metals, metallic materials tend to transform into more thermodynamically stable oxide or salt phases following manufacturing and forming operations, which inevitably promotes oxidative degradation in accordance with the laws of entropy^1,2^. This not only shortens the service life of components but also accelerates the consumption of primary natural resources, consequently disrupting the ecological life cycle. Large-scale studies evaluating the economic impact of corrosion have reported losses equivalent to approximately 2–5% of national GDP values^3–5^. According to the NACE International IMPACT report (2016), the global annual cost of corrosion was estimated to be USD 2.5 trillion, accounting for 3.4% of the world’s GDP, and up to 15–35% of these losses could be mitigated by appropriate corrosion control strategies^2^. These findings highlight that corrosion is not solely an engineering challenge, but rather a systemic issue directly linked to sustainability, energy efficiency, and circular economy principles^1,5^.

To address corrosion challenges in harsh environments, the development of materials with superior corrosion–oxidation stability has continued since the early 20th century. Among advanced material classes brought to industrial scale, nickel-based superalloys introduced in the 1950s have gained considerable attention due to their superior performance in extreme operating conditions^6^. Superalloys are generally classified into nickel-, cobalt-, and iron-based groups, with nickel-based systems being the most widely used owing to their favorable cost-to-performance balance^6,7^.

Among nickel-based superalloys, Inconel 718 (IN718) holds strategic importance in aerospace, power generation, nuclear, marine, and chemical industries due to its excellent high-temperature/creep strength, fatigue resistance, corrosion resistance, and good weldability^7–9^. Its mechanical strength primarily depends on γ″ (Ni₃Nb) precipitates and partially on γ′ (Ni₃(Al, Ti)), while δ (Ni₃Nb) formation and carbide phases (MC, M₆C) govern microstructural stability during service^8,10^. In addition, high Cr and Mo contents promote the formation of Cr₂O₃–MoO₃ enriched passive films that ensure strong localized corrosion resistance even in chloride-containing environments^8^.

However, conventionally cast or wrought IN718 components face limitations when intricate internal channels or topology-optimized geometries are required. These constraints have driven the widespread integration of Additive Manufacturing (AM), particularly Selective Laser Melting (SLM) / Laser Powder Bed Fusion (LPBF), for producing complex IN718 components with improved material utilization and near-net-shape capabilities^11–13^. SLM enables the formation of ultrafine dendritic cell structures through extremely rapid cooling rates (10⁵–10⁷ K/s), but also introduces microstructural heterogeneities such as Laves phase ((Ni, Cr, Fe)₂(Nb, Mo, Ti)), Nb-rich segregations, residual stresses, and micro/nano-porosity, which can compromise passive film stability and increase the susceptibility to localized corrosion, particularly pitting and crevice attack^11,14^. Furthermore, build orientation-dependent texture and grain boundary distribution can modify both mechanical response and corrosion behavior in 3.5% NaCl environments^13^. Therefore, post-processing routes are essential to enhance the mechanical integrity and corrosion performance of SLM-fabricated IN718.

Although several post-treatments such as solutionizing and aging, stress-relief annealing, HIP, dual-aging, shot-peening, electropolishing, and laser surface modification have demonstrated improvements in porosity reduction and passive film continuity^8,11,14–17^, these approaches alone may not sufficiently suppress the synergistic effect of corrosion and wear under harsh service conditions. Recent studies reported that HIP + DA treatment improved microstructural homogeneity and corrosion impedance by converting columnar grains into equiaxed structures^17^, while AM-HIP processing led to superior corrosion resistance compared with as-built and wrought counterparts^8^. Electropolishing has also been shown to enhance pitting potential by removing surface asperities and improving passive film stability^14^. Besides these post-processing methods, thermal spraying and cold spraying are also used in industry for surface performance enhancement^18,19^. Thermal spraying methods are preferred due to their high deposition efficiency, scalability, and suitability for large surface area coating, while cold spraying has significant advantages such as low oxidation, low thermal degradation, and better preservation of feedstock chemistry characteristics because of the lower temperature involved in the process^18,19^. However, these coating processes might still encounter issues such as coating porosity, interfacial bonding, residual stresses, and requirement of post-processing based on the substrate and coating combination. Therefore, alternative or complementary surface engineering strategies that can provide improved microstructural stability and enhanced high-temperature oxidation and corrosion resistance remain of considerable scientific and technological interest.

Considering sustainability and life cycle, the integration of surface engineering and coating techniques with SLM-fabricated IN718 can offer an attractive solution for improving structural integrity and environmental sustainability. In particular, thermochemical surface coatings like boronizing, aluminizing, and boroaluminizing, as well as PVD/CVD and laser cladding, can provide a synergistic benefit to surface hardness and barrier properties to ionic transport, thereby reducing wear-corrosion coupling synergies^20–22^. In particular, thermochemical surface coatings can offer advantages in terms of low capital costs in equipment and ease of application^21^. Although numerous investigations have shown the effectiveness of thermochemical surface coatings like aluminizing or boronizing in improving corrosion and wear resistance of IN718^9,23–31^, there have been relatively fewer investigations involving simultaneous boroaluminizing treatments^31,32^. In particular, in the Ni-Al-B system, dense and adhesive aluminide (Ni₃Al, Ni₂Al₃) and boride (Ni₂B, NiB, Ni₄B₃) phases can form, thereby restricting electron and ion transport while maintaining passive film integrity under high oxidation-chloride environments^31–34^.

Thermochemical surface treatments like aluminizing, boronizing, and boroaluminizing have been found to significantly improve oxidation resistance, corrosion resistance, and wear resistance in IN718 and other high-temperature alloys^9,23–33^. Lei et al.^31^ have shown that high-temperature oxidation resistance in boroaluminized IN718 alloys is superior to that in uncoated samples. Tang et al.^32^ have also shown that corrosion and wear resistance in boroaluminized IN718 alloys are superior. In another study, Günen and Ergin^33^ have shown that thermochemically coated cobalt-based Haynes 25 high-temperature alloys have superior high-temperature wear resistance.

The research gap in this study is not in the thermochemical surface treatment process but in the limited number of studies that have used SLM-produced IN718 to comparatively investigate different surface engineering techniques and their effect on high-temperature oxidation and corrosion resistance. Günay and Günen^34^ investigated the microstructure, hardness, and high-temperature wear resistance (25–750 °C) of SLM-produced IN718 alloys that have been subjected to different thermochemical surface treatments like single-stage boronizing, single-stage aluminizing, sequential aluminizing/boronizing, sequential boronizing/aluminizing, and co-precipitated boroaluminizing. They have shown that the wear resistance in boroaluminized samples was superior to that in samples subjected to other surface treatments. These multiphase structures combine the hardness and chemical stability of borides with the oxide stability of aluminides, providing an alternative to conventional coatings and possibly avoiding the problems of brittleness and adhesion of single-phase coatings.

Nevertheless, a direct comparison of the effects of various thermochemical surface treatments applied to additively manufactured IN718 under the same process conditions has not been adequately addressed in the existing body of literature. In particular, the efficiency of aluminizing (SSA), boronizing (SSB), simultaneous boroaluminizing (CBA), as well as sequential aluminizing-boronizing (SAB) and boronizing-aluminizing (SBA) surface treatments in enhancing the corrosion resistance of SLM-produced IN718 has not been adequately clarified. The primary objective of the current study is to comparatively investigate the effect of various surface treatment approaches on the corrosion resistance of SLM-produced IN718 in a 3.5 wt% NaCl solution. To achieve this objective, surface treatment approaches were conducted under the same conditions, and the coating structure, phase formation, as well as the corrosion response, were examined. The results provide new insights into the correlation between coating structure and corrosion resistance for nickel-based superalloys produced using additively manufactured techniques.

## Materials and methods

The SLM Inconel 718 sample was fabricated on an EOS M290 device using 360 W laser power and 1400 mm/s scanning speed at a base temperature of 80 °C in an argon atmosphere. The bidirectional X-scan strategy, applied with a layer thickness of 40 μm and a hatch spacing of 80 μm, was carried out to ensure single-crystal-like texture formation on the sample as reported in our previous study^35^. The chemical composition of the SLM Inconel 718 sample was determined as 51.25 Ni, 18.77 Cr, 3.8 Mo, 6.28 Nb, 1.14 Ti, 0.1 Co, with the remaining Fe and impurities.

The surface roughness of the specimens to be coated, which are originated from SLM, was eroded by 120 grit SiC abrasive paper in the FORCİPOL 2 V brand automatic sanding machine, which will eliminate from the surface (to a depth of ~ 40–50 μm). Afterwards, the specimens were grouned with 240–400-800, and 1000 grit SiC abrasive papers and the surface was cleaned well with sterile water. Then the samples were cut in the dimensions of 25 × 25 × 5 mm with the METACUT 251 brand sensitive cutting device. Considering 870 °C as the δ-phase transformation temperature for Inconel^36,37^ and based on previous boronizing and boroaluminizing studies, the coating processes were carried out at 980 °C under the conditions detailed in Table 1 below^9,31–34,38^. After the coating processes, the samples were left to cool in the open air.

**Table 1:** Coating process parameters and sample nomenclature^34^.

Naming	Temperature(°C)	Duration (h)	Powder (wt%)
Inc718	As-built	-	-
SSA	980	1	65Al_2_O_3_+30Al+5NH_4_Cl
SAB	980	1	65Al_2_O_3_+30Al+5NH_4_Cl
	980	1	50Al_2_O_3_+45 B_4_C+5NaBF_4_
CBA	980	2	50Al_2_O_3_+30B_4_C+10Al+5NH_4_Cl+5NaBF_4_
SSB	980	1	50Al_2_O_3_+45 B_4_C+5NaBF_4_
SBA	980	1	50Al_2_O_3_+45 B_4_C+5NaBF_4_
	980	1	xx65Al_2_O_3_+30Al+5NH_4_Cl

Coated samples were cut with dimensions of 10 × 10 × 5 mm for metallographic characterization and XRD analysis. For microstructural examination, the samples were hot-mounted, then mechanically grounded using 120–2500 grit SiC sandpaper, and polished sequentially with 3 μm, 1 μm, and 0.25 μm Al₂O₃ suspensions. After polishing, the samples were ultrasonically cleaned in distilled water at a frequency of 20 kHz for 20 min to completely remove surface residues and dried with hot air.

The microstructural morphology and elemental distribution of boride, aluminide, and boroaluminide coatings were analyzed using a Thermo Fisher Scientific Apreo S LoVac SEM system with an integrated EDS detector. A CBS (Circular Backscatter) detector was selected for detailed evaluation of the coating layers, and analyses were conducted at 10 kV accelerating voltage and a 10 mm working distance. The depth profiles of Ni, Cr, Fe, Nb, B, Al, and O were obtained using EDS region scans, and the coating layer, transition zone, and matrix regions were identified using these data. Coating thicknesses were calculated as the average of 50 measurements taken with SEM image analysis software.

The phase compositions and crystallographic properties of the coatings were determined at room temperature using a Rigaku SmartLab XRD system (Cu Kα, λ = 1.542 Å; 40 kV, 30 mA). Diffraction patterns were recorded in the 10°–90° range, at a scan rate of 2°/min, and a 0.02° step width. A 0.8° grazing incidence configuration was also applied to identify low-concentration boride phases. The obtained XRD data were evaluated by quantitative phase analysis using PDXL software, and the phases were identified using a library-based matching method.

The surface porosity of the samples was quantitatively evaluated through digital image processing using the ImageJ (NIH, USA) software. To ensure high-fidelity measurements and minimize artifacts arising from noise and phase contrast, a multi-ROI (Region of Interest) and statistical thresholding approach was employed. The analysis was specifically focused on isolated regions of the surface micrographs, where a minimum of 10 randomly selected ROIs were analyzed to ensure statistical significance. The porosity was defined by isolating the darkest pixel population, representing the bottom ~ 2% of the intensity histogram, to accurately capture micro-cavities while excluding surface oxidation or shading effects. The final porosity value was calculated as the area percentage (Area%) and reported as a mean with standard deviation.

The surface roughness (R_a_) of the specimens was quantitatively characterized using a Huvitz HDS-5800 3D profilometer (Republic of Korea). To ensure a comprehensive evaluation of the surface topography, 2D profiles were generated by scanning a total evaluation length of 4.8 mm at a constant scanning speed of 0.1 mm/s. The average surface roughness (R_a_) was calculated from these 2D profiles in accordance with international standards. To ensure statistical reliability and representativeness of the surface condition, the final Ra values for each sample were determined by averaging 10 independent measurements taken from different regions.

The corrosion behavior of as-built SLM Inconel 718 and thermochemically coated SLM Inconel 718 samples was evaluated using open circuit potential (OCP) and potentiodynamic polarization (Tafel) tests in a 3.5% NaCl solution at room temperature. OCP and Tafel measurements were performed in accordance with ASTM G106 and ASTM G5 standards. OCP measurements were used to determine the corrosion potential (Ecorr), while Tafel measurements were used to calculate the corrosion current density (Icorr) and corrosion rate. During the electrochemical tests, the sample surface area in contact with the solution was kept constant at 1.5 cm². All electrochemical experiments were conducted with a CHI 608E potentiostat/potential analyzer using a conventional three-electrode system installed in a Pyrex glass chamber. The samples were used as working electrodes, a platinum wire as counter electrodes, and an Ag/AgCl (saturated KCl) electrode as reference electrodes^39^. Tafel tests were performed in the potential range of − 250 mV to + 250 mV according to OCP, using a scan rate of 0.1 mV/s. Following completion of the corrosion tests, the samples were ultrasonically cleaned in acetone to remove surface corrosion products and then characterized using field emission scanning electron microscopy (FE-SEM) equipped with EDS to examine surface morphology and elemental changes.

## Results and discussion

### Microstructural characterization

**Fig. 1:**
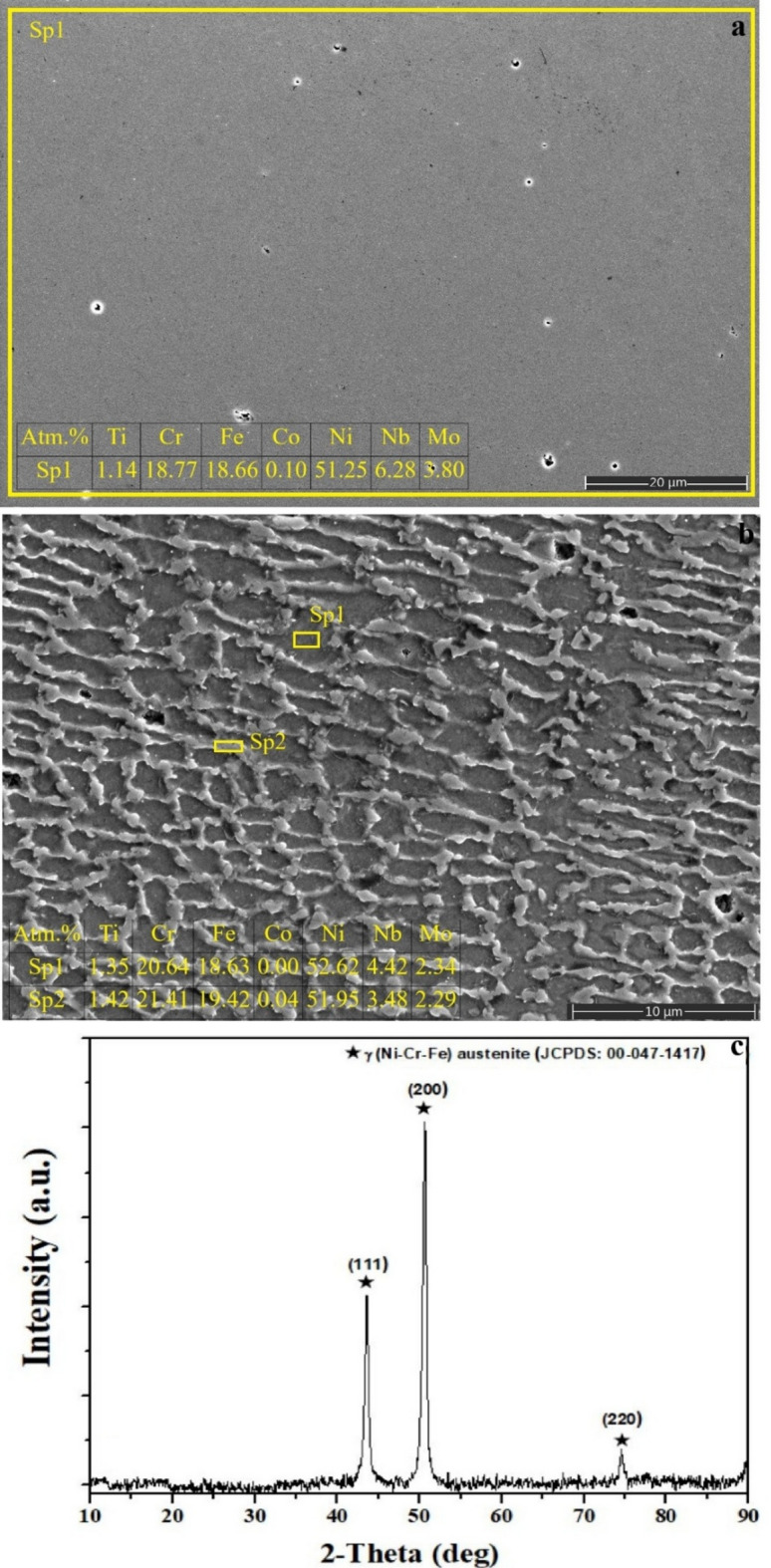
As-built SLM Inconel 718 **a** surface SEM image and EDS analysis, **b** cross-sectional image of the sample and EDS analysis of selected regions, **c** XRD phase analysis.

**Fig. 2:**
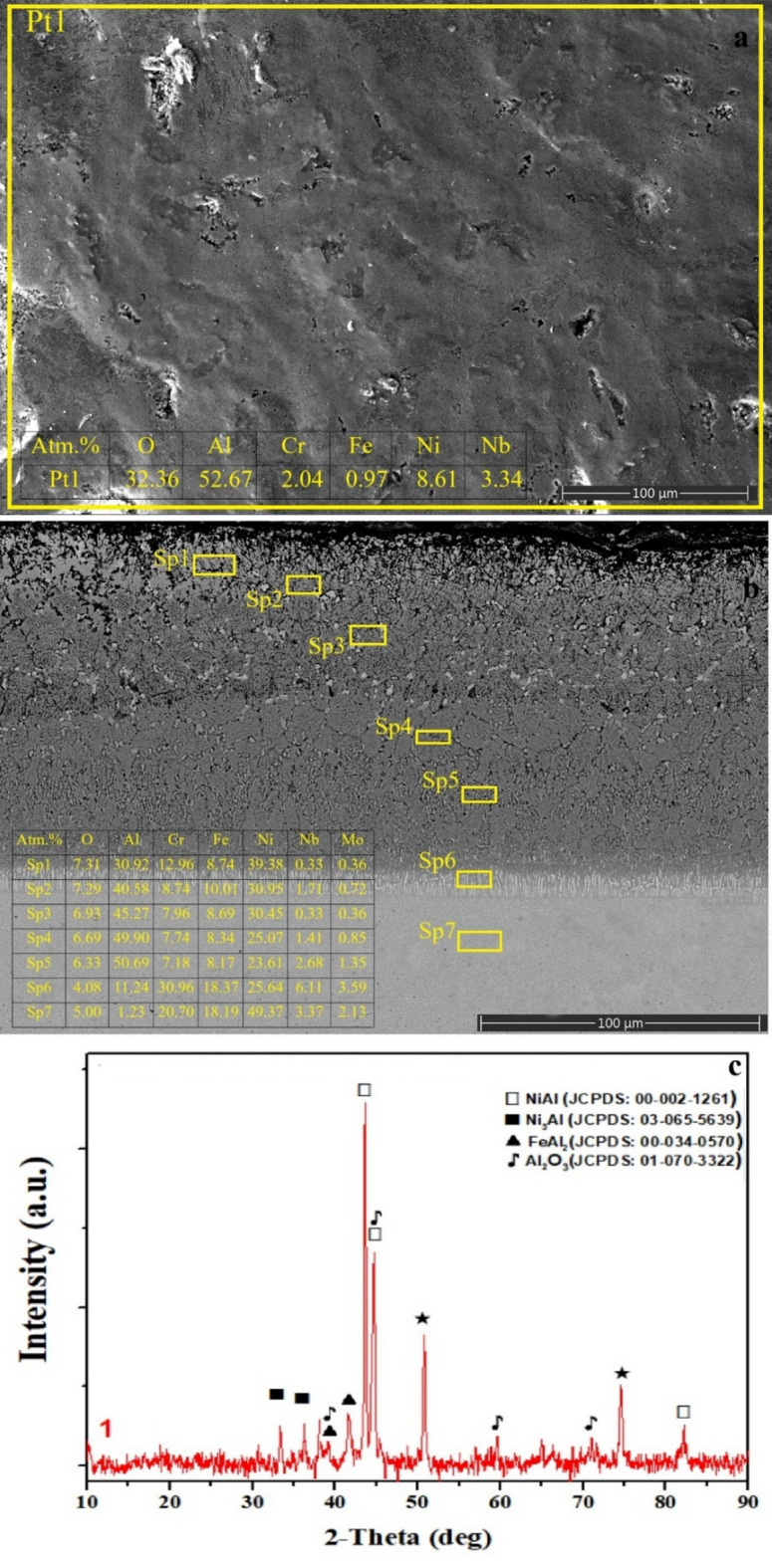
**a** surface SEM image and EDS analysis, **b** cross-sectional view extending from the coating layer to the matrix and EDS analysis of selected regions, **c** XRD phase analysis of SSA sample.

**Fig. 3:**
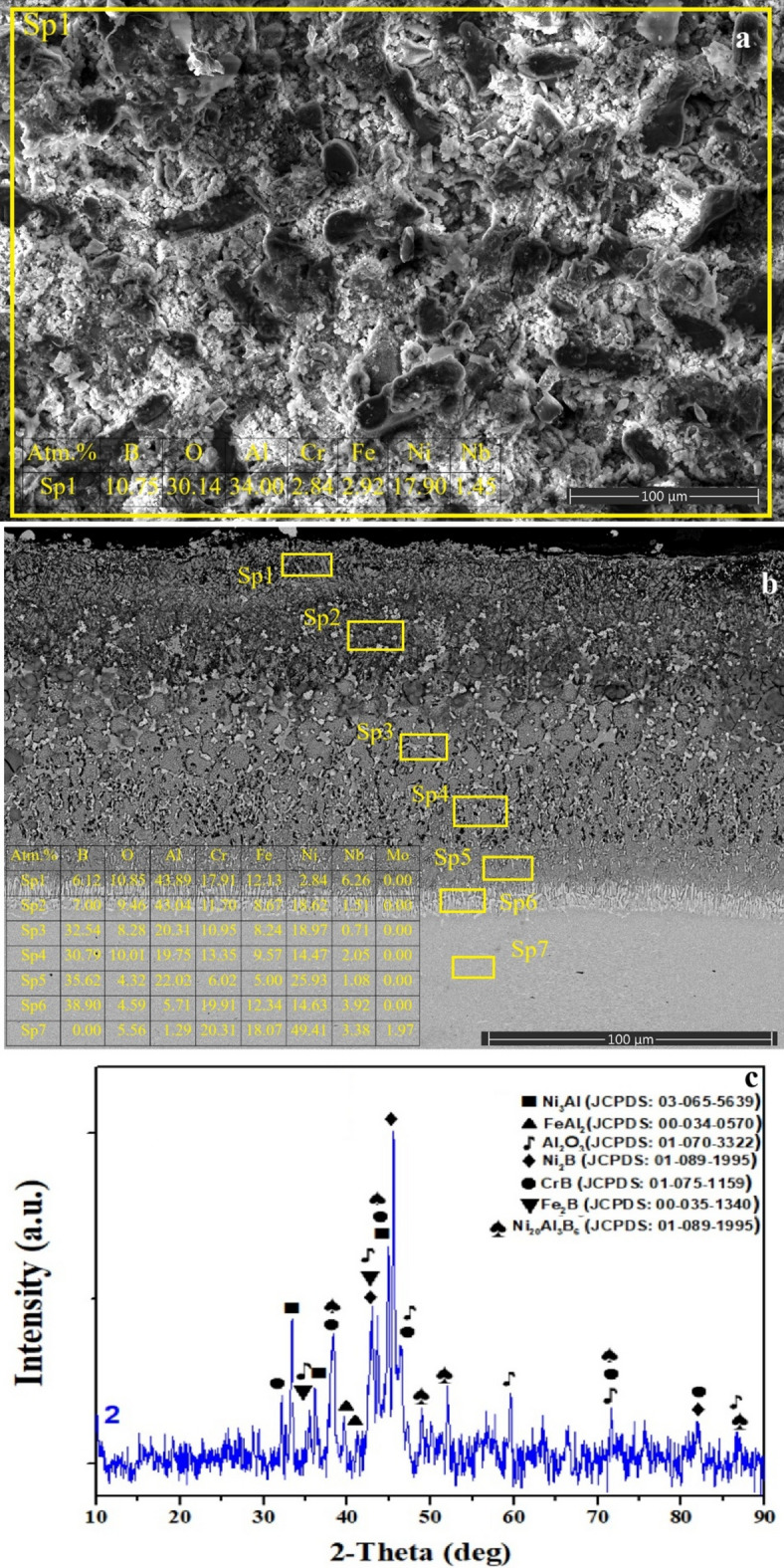
**a** surface SEM image and EDS analysis, **b** cross-sectional view extending from the coating layer to the matrix and EDS analysis of selected regions, **c** XRD phase analysis of SAB sample.

**Fig. 4:**
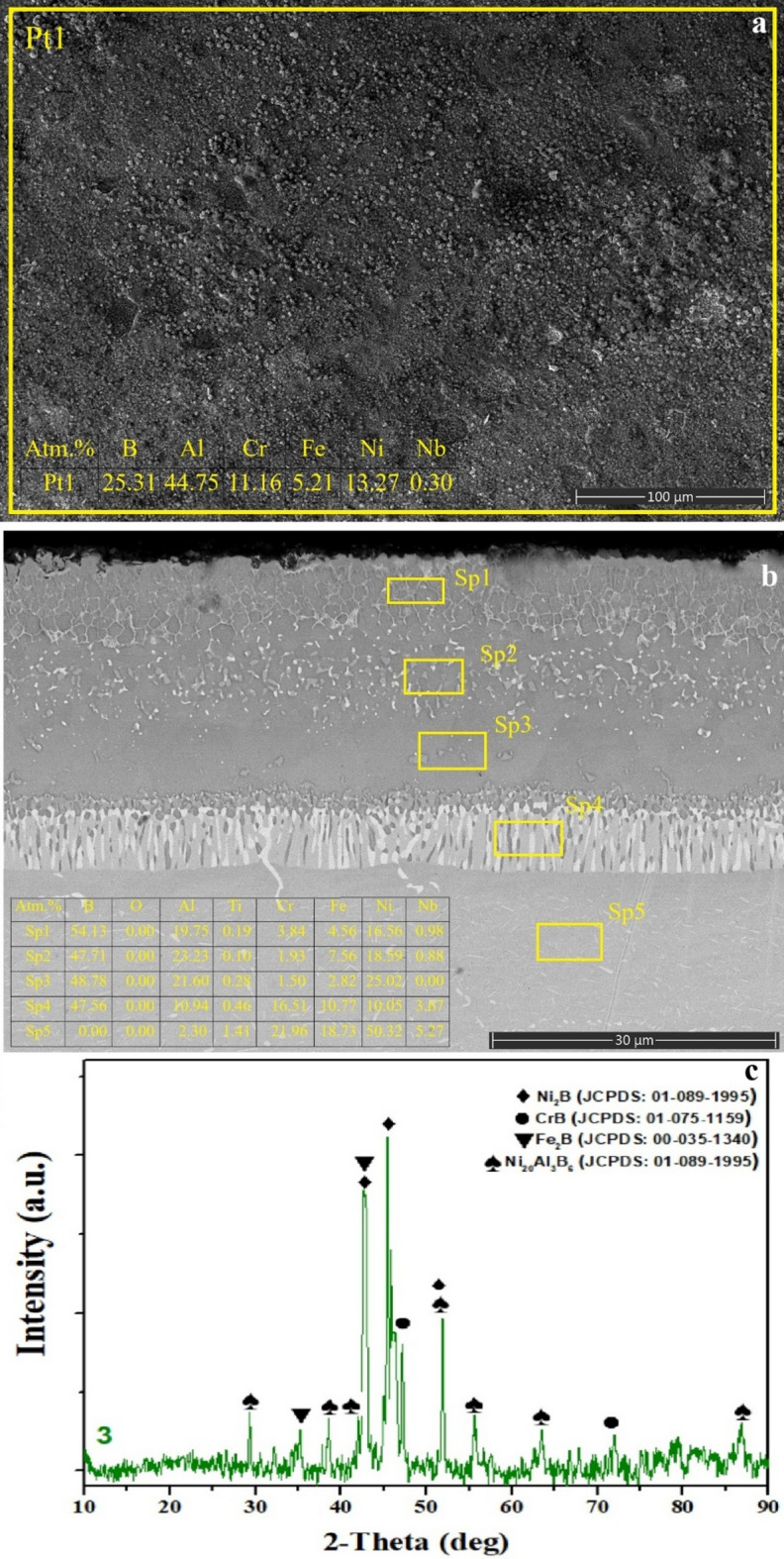
**a** surface SEM image and EDS analysis, **b** cross-sectional view extending from the coating layer to the matrix and EDS analysis of selected regions, **c** XRD phase analysis of CBA sample.

**Fig. 5:**
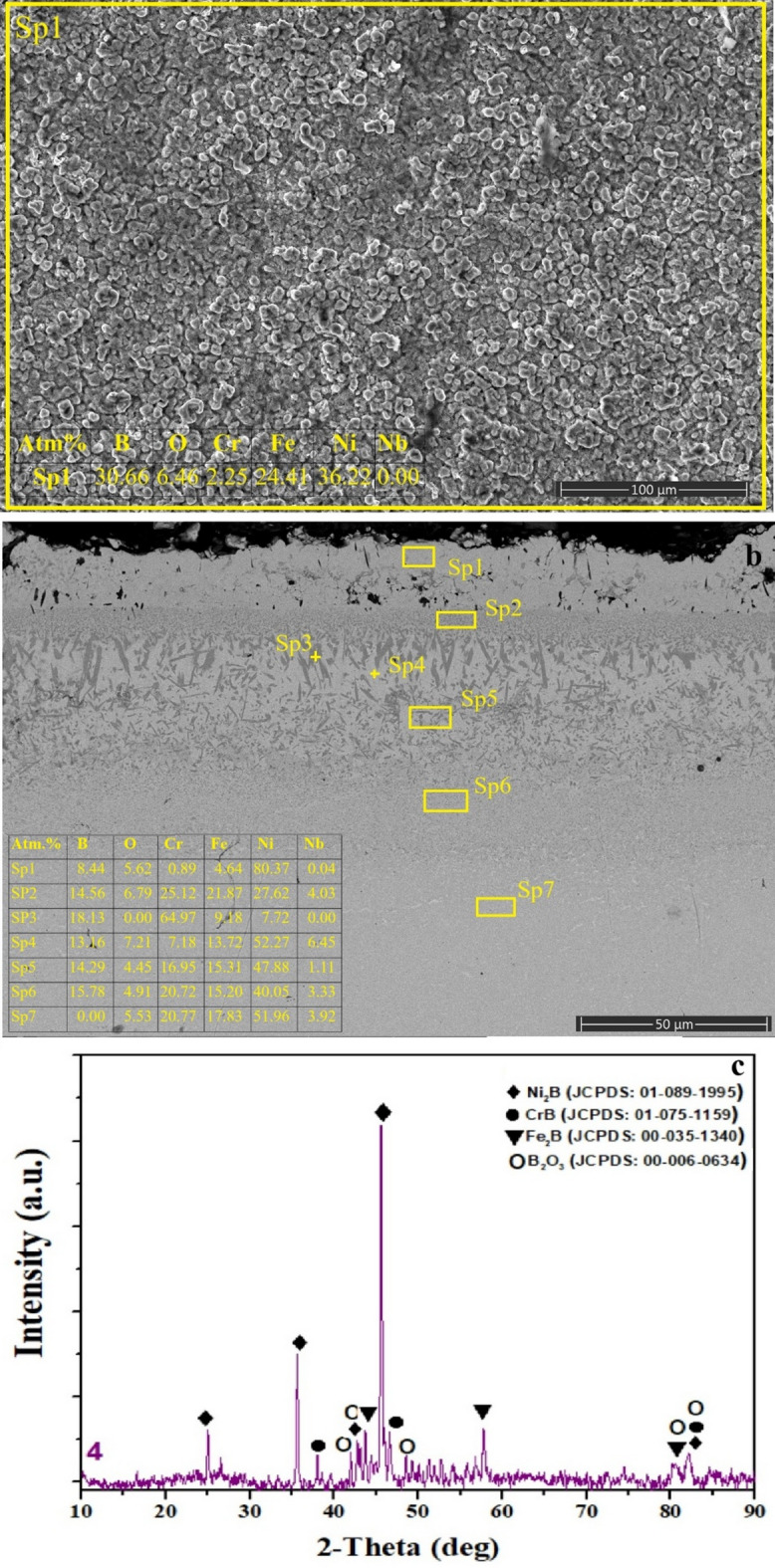
**a** surface SEM image and EDS analysis, **b** cross-sectional view extending from the coating layer to the matrix and EDS analysis of selected regions, **c** XRD phase analysis of SSB sample.

**Fig. 6:**
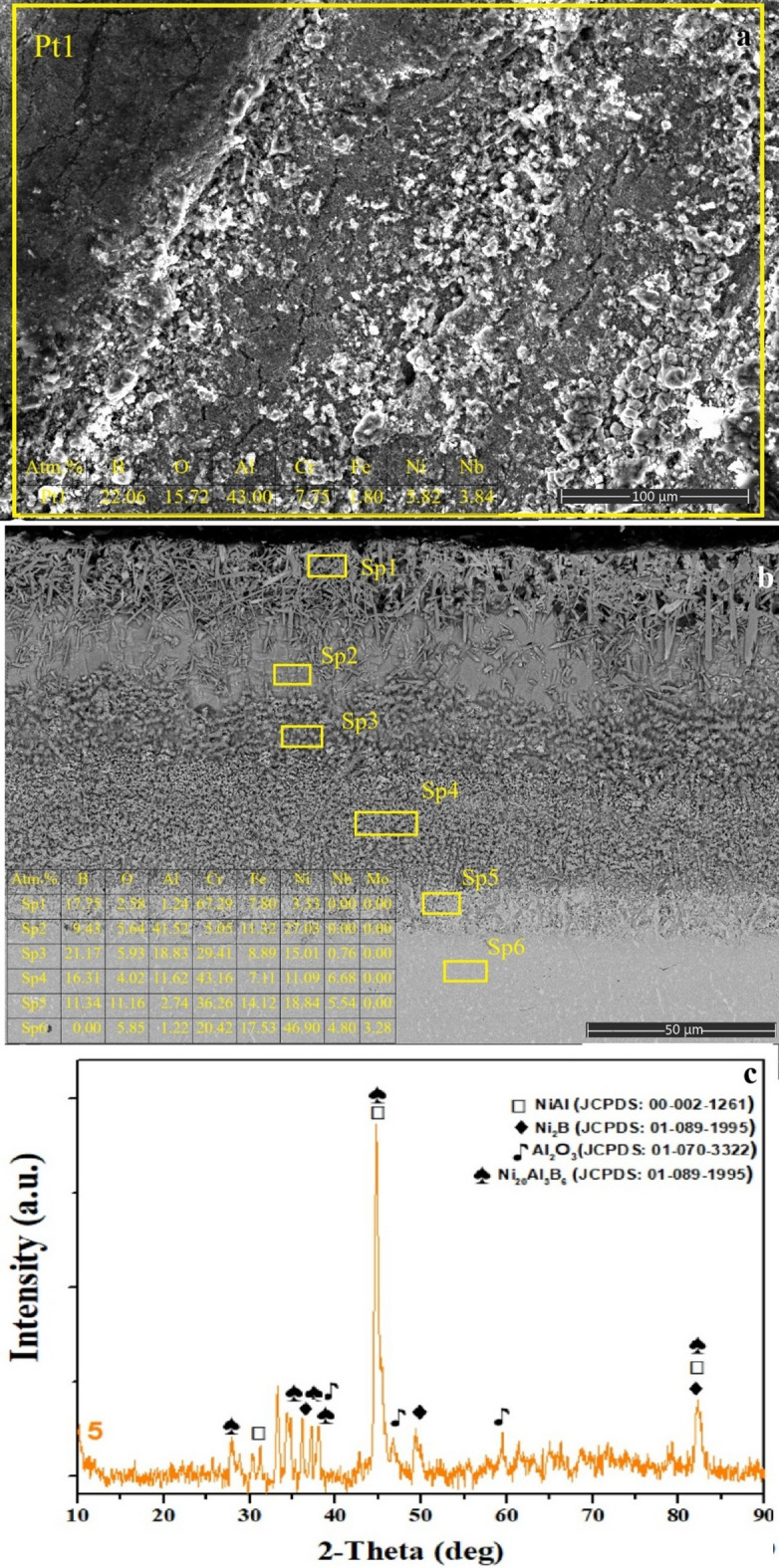
**a** surface SEM image and EDS analysis, **b** cross-sectional view extending from the coating layer to the matrix and EDS analysis of selected regions, **c** XRD phase analysis of SBA sample.

Figures 1, 2, 3, 4, 5, 6 presents surface and cross-sectional SEM-EDS analyses and XRD analyses of the samples after as-built and different coating routines.

The polished surface of the as-built SLM Inconel 718 exhibits a relatively smooth morphology with localized pit-like micro-cavities (Fig. 1a). These features are characteristic voids generated by partially unmolten powder particles adhered to the surface during the SLM process. Similar morphologies have been reported in the literature and are attributed to un-molten particles and/or insufficient interlayer fusion, commonly referred to as lack-of-fusion defects^40–42^. The EDS results (Ni ≈ 51 at%, Cr ≈ 19 at%, Fe ≈ 19 at%, Nb ≈ 6 at%) confirm that the analyzed surface corresponds purely to the alloy matrix, indicating the absence of surface oxidation or foreign coating residues. This verifies that the polishing process effectively removed any oxidized layer and that the observed cavities originate solely from SLM-induced particle adherence.

The cross-sectional SEM micrograph (Fig. 1b) clearly reveals a typical columnar–dendritic microstructure formed due to the rapid solidification characteristic of the SLM process. Thin dendrite arms aligned parallel to the laser scanning direction indicate extremely high cooling rates^40^. Moreover, the presence of rounded and oval pores throughout the structure corresponds to trapped gas pockets and/or unmolten powder inclusions, representing inherent defects arising from the layer-wise fabrication nature of SLM^40,43^.

EDS performed at the Sp1 region represents the γ-matrix phase, showing a composition consistent with the nominal chemistry of Inconel 718 in terms of Ni, Cr, and Fe. The Sp2 region, analyzed from dendritic zones, exhibits slightly higher Cr and Fe levels, while Nb and Mo show minor segregation along dendrite arms—an indication of micro-segregation due to the high solidification rate in SLM^43^. The relatively low degree of chemical segregation is consistent with the XRD results, which only revealed a single γ-(Ni-Cr-Fe) austenitic phase. The XRD pattern shown in Fig. 1c confirms the presence of the γ-matrix through strong, sharp reflections corresponding to the (111), (200), and (220) planes, indicative of a fine-grained, directionally solidified dendritic structure typical of SLM-processed IN718. The absence of any intermetallic phase reflections (Laves, δ, γ′)^44^ confirms that the adopted SLM parameters stabilized the γ-matrix as the dominant phase.

The surface SEM image of the SSA sample (Fig. 2a), together with point EDS analysis, reveals the formation of a distinctly Al–O enriched outer layer after the aluminizing process^45^. The high Al (≈ 52.7 at%) and O (≈ 32.3 at%) contents measured at Pt1 confirm the predominance of Al₂O₃ on the surface. This observation is fully consistent with both the elevated Al and O concentrations detected in the cross-section (Fig. 2b) and the characteristic Al₂O₃ diffraction peaks identified in the XRD pattern (Fig. 2c). The continuity of the Al₂O₃ layer and its relatively low Ni and Fe contents indicate that the aluminizing treatment conducted in ambient atmosphere promotes oxidation-driven surface reactions, resulting in a stable protective oxide barrier^34^.

A detailed examination of the cross-section (Fig. 2b) shows that the EDS profiles acquired at Sp1–Sp5 exhibit a gradual variation in elemental distribution through the coating thickness: Al content increases with depth, while oxygen content decreases progressively. The consistently elevated Al contents across these regions suggest the formation of NiAl/Ni₃Al intermetallics and Al₂O₃ due to interdiffusion and reaction between Al, Ni, and oxygen. The presence of NiAl (B2), Ni₃Al (γ′), and Al₂O₃ diffraction peaks in the XRD pattern further confirms the development of a multiphase aluminide diffusion layer (Fig. 2c).

At deeper regions (Sp6), Al concentration decreases while Fe and Ni levels increase, indicating the formation of Fe-rich aluminide phases such as FeAl₂, as supported by XRD analysis. Although Cr concentration is also relatively elevated at this depth, no Cr–Al related phases were detected in the diffraction pattern. The gradual compositional transition from the oxide-rich surface toward the substrate is characteristic of a well-defined interdiffusion layer (IDL) typical of aluminide coatings^45^. It is also considered that the high density of grain boundaries inherent to SLM microstructures enhances local diffusion rates, thereby promoting coating growth and compositional grading.

Following the SAB process, the surface exhibits a distinctly porous and heterogeneous morphology (Fig. 3a). Surface EDS analysis reveals high concentrations of Al (≈ 34 at%), O (≈ 30 at%) and B (≈ 10.8 at%), indicating the development of Al₂O₃ together with boride and aluminide phases formed through intensive surface reactions during sequential aluminizing and boronizing. The observed micro-voids are likely associated with localized vacancy formation caused by diffusion fluxes and gaseous reaction products generated during boronizing, as similarly reported elsewhere^31^.

The compositional gradients through the coating thickness (Fig. 3b) show that Al and O contents are high in the near-surface region and decrease gradually toward deeper layers. Conversely, boron exhibits the opposite trend; relatively lower contents are detected near the surface (Pt1, Pt2), while higher levels appear in the subsurface (Pt3, Pt4). Elevated Al, B, and O amounts at Sp1–Sp5 suggest the formation of multiple reaction products, including NiAl, Ni₂B, Fe₂B, and Al₂O₃, within the outer diffusion zone of the SAB coating. At deeper regions (Sp6), a further reduction in Al and a noticeable increase in Ni and Fe indicate a transition into a typical boron–aluminide diffusion zone (BA-DZ).

Notably, the high B concentrations at Sp4–Sp6 (≈ 30–38 at%) imply that boron, owing to its small atomic radius, readily diffuses to deeper levels, thereby promoting the formation of Fe₂B and CrB phases in these subsurface regions. This interpretation is supported by the XRD pattern (Fig. 3c), which confirms the coexistence of multiple phases. Besides borides such as Ni₂B, Fe₂B, and CrB, aluminide phases including NiAl and FeAl₃ are also identified, providing clear evidence of sequentially induced aluminide–boride formation during the SAB treatment^33,34^. Furthermore, the presence of strong Al₂O₃ peaks suggests that oxidation during processing in ambient atmosphere contributes to the formation of a surface oxide layer; however, this oxide formation does not hinder the development of underlying boron–aluminide layers.

Figure 4a shows that the surface obtained after the CBA treatment exhibits a more compact and less porous morphology compared to the porous structures observed in the SSA and SAB samples. Surface EDS results indicate high Al (≈ 45 at%) and B (≈ 25 at%) concentrations, confirming that simultaneous diffusion of aluminum and boron during the CBA process promotes the intensive formation of mixed boron–aluminide phases (Fig. 4a, EDS). The simultaneous enrichment of Al and B suggests favorable conditions for the formation of Ni₂B, Fe₂B, and complex NixAlyBz-type phases on the surface^31,33,34^.

Cross-sectional SEM and EDS analyses (Fig. 4b) show that the diffusion layer produced by CBA is more compact and uniform than that of SAB, although it is comparatively thinner. The composition measured at Sp1–Sp3, where B contents remain within ≈ 47–54 at% and Al within ≈ 19–23 at%, indicates the development of a relatively homogeneous boroaluminide layer throughout the coating thickness. At Sp4–Sp5, an increase in Cr and Fe with lower Al contents and nearly unchanged B levels suggests a transition into a diffusion zone dominated by CrB and FeB-type borides. This structure confirms the characteristic multilayered boron–aluminide diffusion layer that forms as a result of the simultaneous reaction mechanism inherent to CBA processing.

The XRD pattern (Fig. 4c) supports these microstructural findings by revealing not only dominant Ni₂B, Fe₂B, and CrB peaks but also metastable complex boroaluminide phases such as Ni₂₀Al₃B₆. This demonstrates that the CBA process promotes a more uniform and compositionally enriched boroaluminide phase formation compared to SSA and SAB. The detection of boroaluminide phases together with the consistent Al/B ratios observed in the EDS profiles confirms the successful simultaneous incorporation of both boron and aluminum, effectively enhancing the phase architecture of the coating.

The surface morphology formed after the SSB process (Fig. 5a) appears significantly more compact and less porous than that observed in the SAB coating^46,47^, yet still exhibits a higher surface porosity and a rather flaky texture compared to the CBA coating. The surface EDS analysis shows high boron content (≈ 30.7 at%) together with a moderate oxygen level (≈ 6.5 at%), indicating the presence of boride phases (Ni₂B, Fe₂B, CrB) detected in XRD results^9,23–26,30–32,34^, along with localized B₂O₃ residues formed due to oxidation reactions occurring during the initial stages of boronizing. The flaky surface pattern can be attributed to volumetric stresses induced during boron diffusion and surface cracking driven by oxidation^45^.

The cross-sectional SEM image (Fig. 5b) reveals a well-defined multilayered boride structure. At the outermost Sp1 region, the relatively low B content (≈ 8.4 at%) accompanied by high Ni levels (≈ 80.4 at%) and minor oxygen (≈ 5.6 at%) suggests a slightly oxidized boron-depleted region near the surface. In Sp2, a notable increase in B content (≈ 14.56 at%) and a rise in Cr/Fe levels indicates the initiation of boride formation where boron begins to react strongly with the matrix. Sp3–Sp5 represent the main boride layer, characterized by high B concentrations (≈ 13–18 at%) and pronounced Cr enrichment (e.g., Sp3: Cr ≈ 65 at%). The dark gray zones observed at Pt3 correspond to Cr-rich regions, suggesting localized precipitation of CrB/Cr₂B phases driven by boron–chromium reactivity. Furthermore, Fe (≈ 13–15 at%) and Ni (≈ 48–52 at%) contents detected at Sp4 and Sp5 confirm the coexistence of Fe₂B and Ni₂B phases. The increasing Ni and Fe levels within this region indicate that mixed Ni₂B–Fe₂B phases dominate the main boride zone. Sp6, where B remains at ≈ 15 at%, reflects the transition to the interdiffusion zone, where boride phases gradually diminish as Ni (≈ 40 at%) and Fe (≈ 15 at%) contents increase, indicating the development of a B-enriched γ-matrix. Finally, Pt7 marks the underlying region where the boride coating structure fully terminates.

Consistent with these microstructural findings, the XRD pattern of the SSB specimen (Fig. 5c) confirms the presence of dominant Ni₂B, Fe₂B, and CrB phases on the surface^9,23–26,30–32^. Additionally, the appearance of low-intensity B₂O₃ reflections provides crystallographic evidence of the surface oxidation behavior observed.

As shown in Fig. 6a, the SBA-treated surface exhibits a heterogeneous morphology characterized by localized pore formation together with spallation/flaking features^31^. The high Al (≈ 43 at%) and moderate B (≈ 22 at%) concentrations detected at Pt1 indicate that aluminum effectively diffused toward the surface during the second treatment stage, while partial degradation of the previously formed boride layer resulted in microvoids through oxidation- and spallation-driven mechanisms. The coexisting pore–flake morphology suggests that the brittle boride layer formed during boronizing underwent partial delamination under thermal exposure during the subsequent aluminizing step.

The cross-sectional SEM image (Fig. 6b) indicates the formation of a multilayer structure in the SBA coating, consisting of needle-like borides in the outer region and an aluminide-enriched diffusion zone beneath^31–33^. The outermost Sp1 region, with elevated B (≈ 17.8 at%) and Cr contents, corresponds to the preserved Ni₂B-, CrB-, and Fe₂B-type boride layer originating from the initial boronizing process. The needle-like morphology at this location is a characteristic feature of borides formed during boron diffusion. At Sp2, a notable decrease in B (≈ 9.5 at%) accompanied by a significant increase in Al (≈ 42 at%) confirms the dominant formation of aluminide phases, attributed to the delayed aluminizing treatment after boronizing. Across Sp1–Sp4, the presence of both Al and B in varying ratios clearly indicates the development of a boroaluminide layer, albeit with a locally heterogeneous distribution. The Pt5 region, characterized by high B (≈ 11 at%) and reduced Al (~ 3 at%) along with strong Ni enrichment (≈ 47 at%), represents the transition zone, where boron diffusion continues but aluminide involvement diminishes. Finally, Pt6 reveals near-zero Al and B contents, corresponding to the unaffected γ-matrix region of the substrate.

The XRD pattern (Fig. 6c) distinctly verifies the coexistence of aluminide and boride phases in the SBA coating. The detection of NiAl, Ni₂B, and Al₂O₃ peaks confirms that borides formed during the boronizing step are retained after aluminizing, while the strong NiAl reflections demonstrate the successful formation of aluminide phases. Additionally, the presence of complex Ni_x_Al_y_B_z_ boroaluminide phases strongly supports the dual-stage reaction mechanism characteristic of the SBA process.

### Electrochemical evaluation of corrosion performance

**Fig. 7:**
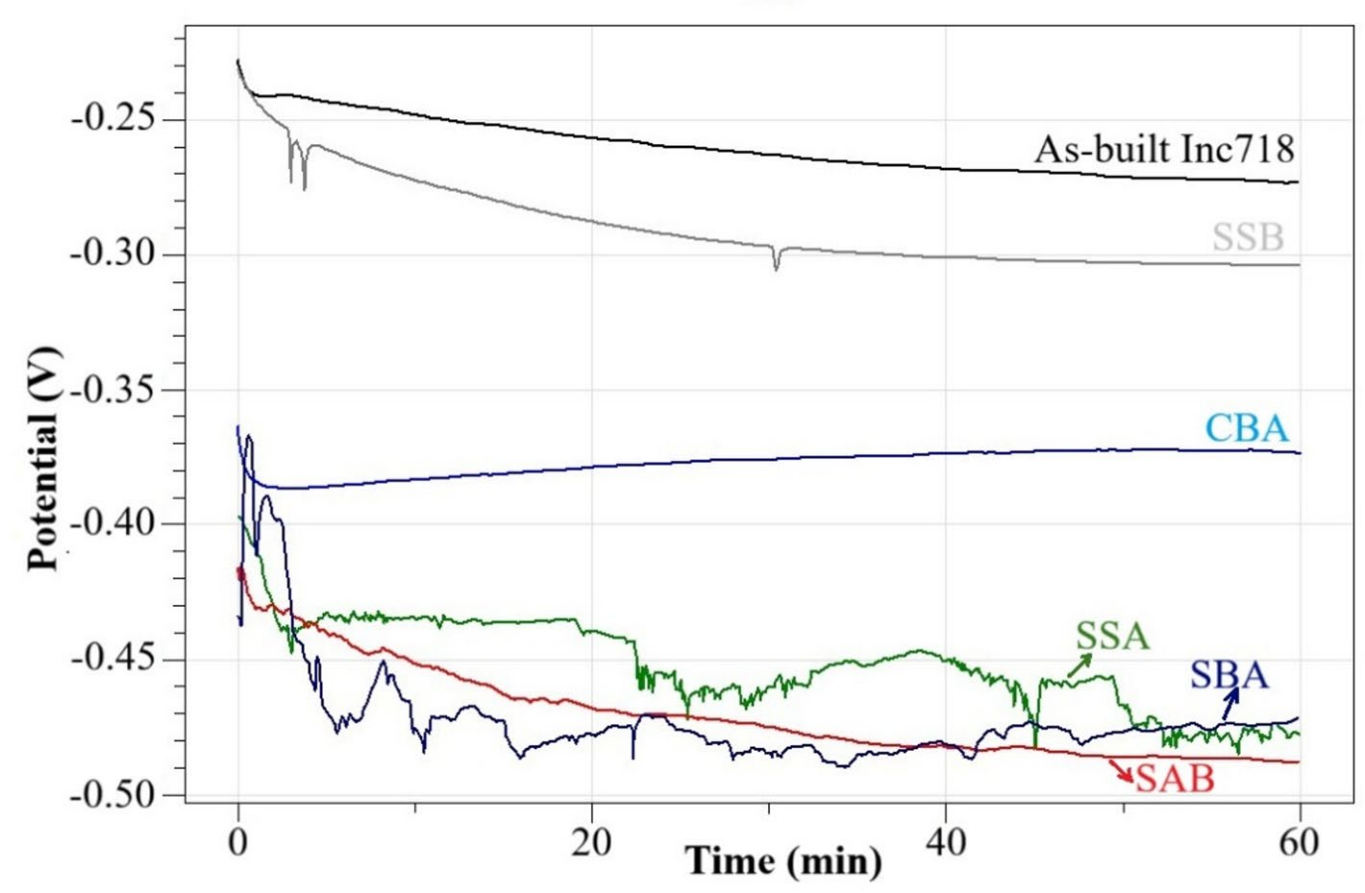
OCP curves of as-built and coated samples in 3.5% NaCl solution.

**Fig. 8:**
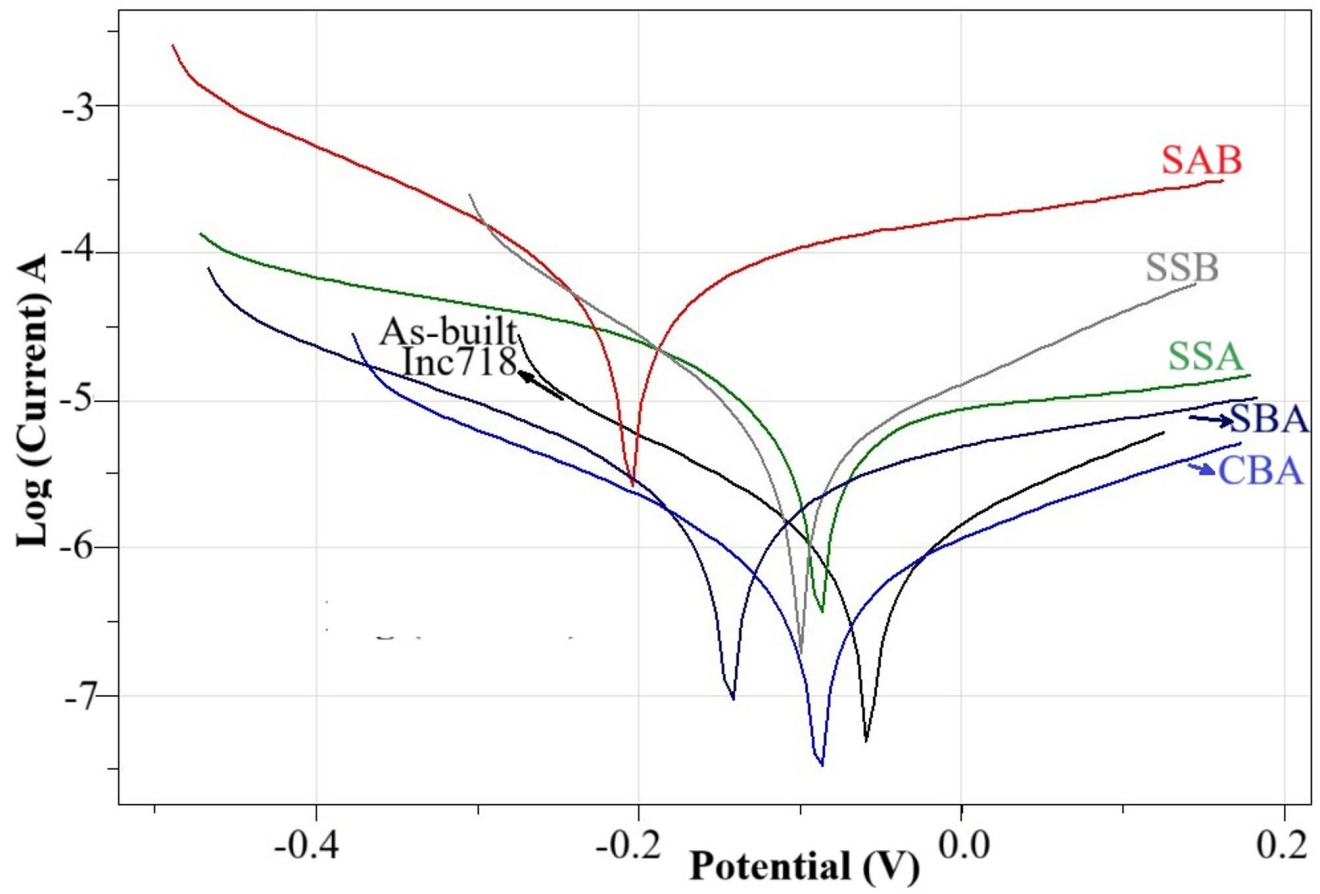
Tafel curves of as-built and coated samples in 3.5% NaCl solution.

Figures 7 and 8 present the OCP and Tafel polarization curves obtained from the corrosion tests of the as-built and coated samples in a 3.5% NaCl solution.

The open-circuit potential (OCP) results demonstrated that the corrosion tendency of Inconel 718 is strongly influenced by the applied surface modification routes. The as-built alloy exhibited the most positive and stable potential (≈ − 0.26 V), which is indicative of a naturally formed and relatively stable passive film on the surface. Thuneman et al. (2024) reported an OCP value of approximately − 0.34 V for SLM-processed Inconel 718 in a 3.5 wt% NaCl solution, confirming the alloy’s ability to form a protective passive layer under chloride-containing environments^8^. In addition, Tang et al. (2022) showed that the passive film generated on SLM-fabricated Inconel 718 in 3.5 wt% NaCl exhibits localized defects, which reduce its resistance to film breakdown and consequently diminish overall corrosion performance^47^. SSB sample showed a slightly more negative potential (≈ − 0.30 V), suggesting that the boride layer provides moderate protection but suffers from micro-defects and porosity^30,48^. CBA sample resulted in a potential of ≈ − 0.39 V with remarkable stability, highlighting the beneficial effect of a homogeneous and integrated surface layer. SSA sample presented more negative potentials around − 0.45 V, accompanied by noticeable fluctuations, which point to discontinuities within the oxide film. In contrast, sequential treatments significantly influenced the corrosion response. Tang et al. (2024) reported that the co-deposited boroaluminizing (CBA) treatment produces a compact and homogeneous multi-phase diffusion layer, which provides enhanced electrochemical stability in 3.5 wt% NaCl solution, evidenced by a more stable open-circuit potential (OCP) and reduced corrosion current densities^32^. Consistently, the present study also demonstrates that the CBA-treated sample exhibits the most stable OCP response among all investigated surface conditions. SAB produced the most negative potential (≈ − 0.48 V), continuously decreasing during the test, reflecting severe galvanic effects and poor passivity. The pore formation on the surface of this specimen was considerably higher compared to the other samples (Fig. 3a). This allowed the corrosive medium to penetrate and accumulate within these pores, increasing the effective contact surface area and consequently leading to a higher corrosion potential^49^. SBA yielded similar negative potentials (≈ − 0.47 V) but with greater stability, indicating that the alumina layer adheres more effectively onto the boride substrate. Therefore, based on the OCP diagrams, the corrosion potentials of the samples can be ranked as as-built > SSB > CBA > SSA ≈ SBA > SAB.

The Tafel polarization curves provide detailed insight into the electrochemical kinetics of the corrosion processes. The as-built Inc718 alloy displayed a moderate corrosion current density, indicating that although its passive oxide film offers partial protection, it is insufficient under aggressive environments^48,52^. The SSA sample demonstrated a clear improvement, with lower current densities compared to the untreated alloy. This enhancement can be attributed to the formation of a stable Al₂O₃ layer, which reduces the anodic dissolution rate^52^. In contrast, SAB saple resulted in the highest corrosion current density among all samples. This outcome is related to the heterogeneous and discontinuous structure of the surface formation, which creates galvanic micro-couples and accelerates corrosion. A similar situation was observed in multilayer PVD coatings by Gaona-Tiburcio et al.^53^. CBA sample produced significantly reduced current densities, showing that the co-deposition process yields a more homogeneous surface film with improved electrochemical stability. This outcome is a direct result of the formation of the most compact surface and the most homogeneous coating layer among the samples, as also evidenced by the surface and cross-sectional examinations (Fig. 4) as stated by Tang et al. 2024^32^. SSB sample exhibited intermediate corrosion behavior: while the boride layer enhances hardness, its porous structure (Fig. 5a) facilitates electrolyte penetration, thereby limiting its corrosion resistance^49^. On the other hand SBA lowest corrosion current density, highlighting the synergistic effect of the two treatments when applied in this sequence. The boride layer provides a mechanically robust foundation, while the aluminized outer surface contributes a dense and protective oxide layer. Therefore, considering the Tafel diagrams, it can be said that the corrosion potentials of the samples are ranked as follows: SBA > CBA > SSA > SSB > as-built Inc718 > SAB.

Considering Figs. 7 and 8 It can be said that although OCP and Tafel analyses are both electrochemical techniques, they emphasize different aspects of corrosion behavior. OCP reflects the thermodynamic tendency of a surface to corrode, representing the equilibrium potential of the system. In contrast, Tafel analysis provides kinetic information, specifically the corrosion current density, which directly correlates with the corrosion rate^54^. In the present study, as-built Inc718 showed the most positive OCP (Fig. 7), suggesting a favorable passive state, yet its Tafel curve indicated a relatively high current density (Fig. 8). On the other hand, SBA sample exhibited one of the most negative OCP values but the lowest corrosion current density in the Tafel test, indicating superior corrosion resistance in kinetic terms. Similarly, SAB showed the poorest performance in both analyses, with highly negative OCP and high Tafel currents. These findings highlight that OCP is primarily useful for assessing passivation tendencies, whereas Tafel analysis provides a more reliable evaluation of actual corrosion resistance. The combined interpretation of both techniques ensures a more comprehensive understanding of the protective efficiency of surface treatments. Table 2 is provided for more quantitative comparison.

**Table 2:** Corrosion values of as-built and thermochemically coated SLM IN718 samples.

Sample	Inc718	SSA	SAB	CBA	SSB	SBA
OCP Start	−0.2282	−0.3968	−0.4209	−0.3634	−0.2315	−0.4331
OCP End	−0.2732	−0.4771	−0.4879	−0.3733	−0.304	−0.4711
E. Corr (V)	−0.0499	−0.099	−0.2244	−0.0714	−0.0921	−0.1478
I. Cor. (A/cm^2)	7.80E-04	3.83E-03	6.15E-02	5.65E-04	3.73E-03	1.73E-06
Rp Ohm	4.74E + 07	8.75E + 03	9.10E + 02	8.35E + 07	7.96E + 03	3.06E + 04
ba (V/dec)	0.195	0.405	0.639	0.253	0.203	0.487
bc (V/dec)	0.18	0.121	0.207	0.227	0.139	0.255
C. Rate (mm/y)	1.86E-02	9.10E-02	1.46E + 03	1.35E-02	8.87E-02	4.11E-02

The electrochemical data obtained from the software analysis (Table 2) highlight the effect of different surface treatments on the corrosion response of SLM Inc718. The as-built sample exhibited an Ecorr of − 0.0499 V and a corrosion current density of 7.80 × 10^− 4^ A/cm², corresponding to a corrosion rate of 1.86 × 10^− 2^ mm/y. This confirms that the naturally formed passive oxide film on the as-built IN718 substrate provides relatively high corrosion resistance, which explains its lower corrosion current density compared with several of the treated samples, as reported in previous works on Ni-based superalloys exposed to chloride media^8,48^. The SSA sample shifted the potential slightly negative (–0.099 V) but decreased the corrosion rate to 9.1 × 10^− 2^ mm/y. The improvement in polarization resistance (Rp ≈ 8.75 × 103 Ω) confirms the protective role of a continuous Al₂O₃ film, consistent with findings that aluminide layers significantly enhance passivation stability in Ni alloys^52^. On the other hand, the SAB sample resulted in the poorest behavior, with a highly negative potential (–0.224 V), a very high current density (6.15 × 10^− 2^ A/cm²), and an extreme corrosion rate of 1.46 mm/y. This can be attributed to the heterogeneity of the layered structure and micro-galvanic effects, which accelerate anodic dissolution. Similar observations were reported for multi layer coatings where discontinuities acted as preferential attack sites^53^. However, CBA sample yielded a relatively noble potential (–0.071 V) and one of the lowest current densities (5.65 × 10^− 4^ A/cm²), giving a low corrosion rate of 1.35 × 10^− 2^ mm/y. This result suggests that the co-deposition process promotes a homogeneous, adherent layer with improved electrochemical stability, which is in agreement with reports on co-deposited aluminide/boride systems^32^. SSB sample showed intermediate results. Although the corrosion rate (8.87 × 10^− 2^ mm/y) was reduced compared to SAB, the Rp value (7.96 × 103 Ω) remained modest. This reflects the inherently porous morphology of boride layers, which, despite their hardness, allow electrolyte penetration. The most favorable performance was observed in the sample subjected to boronizing followed by aluminizing (SBA). This specimen exhibited the highest polarization resistance (3.06 × 10^4^ Ω) and the lowest corrosion current density (1.73 × 10^− 6^ cm²), corresponding to a corrosion rate of only 4.11 × 10^− 2^ mm/y. This result highlights the synergistic effect of the process sequence: a mechanically robust boride sublayer supporting a compact Al₂O₃ top film, producing superior corrosion protection^32,55^. Comparable improvements have been reported in multilayer systems where a hard sublayer enhances adhesion while the oxide film ensures passivation.

**Table 3:** Pre-corrosion and post-corrosion porosity (%) and average surface roughness (Ra) values of the samples.

Sample	Pre-corrosion Porosity (%)	Post-corrosion Porosity (%)	Pre-corrosion (*R*_a_)	Post-corrosion (*R*_a_)
INC718	1.87 ± 0.07	2.45 ± 0.07	0.42 ± 0.04	2.15 ± 0.22
SSA	2.06 ± 0.21	6.82 ± 0.42	1.35 ± 0.12	8.42 ± 1.15
SAB	4.13 ± 0.25	5.15 ± 0.35	4.68 ± 0.55	6.28 ± 0.85
CBA	1.47 ± 0.12	4.30 ± 0.28	2.05 ± 0.20	4.95 ± 0.60
SSB	1.88 ± 0.13	1.92 ± 0.12	2.92 ± 0.35	1.62 ± 0.15
SBA	3.12 ± 0.28	2.18 ± 0.15	3.85 ± 0.45	2.38 ± 0.30

The quantitative surface analysis presented in Table 3 confirms that the electrochemical corrosion resistance of thermochemically coated SLM-Inconel 718 is intrinsically linked to both initial surface roughness (R_a_) and porosity levels. Prior to corrosion testing, the CBA and INC718 samples exhibited the lowest porosity (1.47%) and (1.87%), respectively), which initially suggested a favorable barrier against electrolyte infiltration. However, post-corrosion data revealed a dramatic shift; while the SBA coating maintained its structural integrity with a relatively low post-corrosion porosity of (2.18%), the SSA and SAB samples showed a significant increase in surface degradation, reaching porosity levels of (6.82%) and (5.15%). This degradation is further corroborated by the surface roughness values, where the SBA coating exhibited a stable Ra transition, whereas the SSA sample’s roughness escalated from 1.35 μm to 8.42 μm due to extensive pit-driven surface fragmentation. These findings demonstrate that a compact and chemically integrated boroaluminide layer, as seen in the SBA and CBA treatments, effectively suppresses the electrolyte penetration into SLM-induced defects, thereby minimizing localized anodic dissolution and maintaining surface stability in chloride-containing environments.

**Fig. 9:**
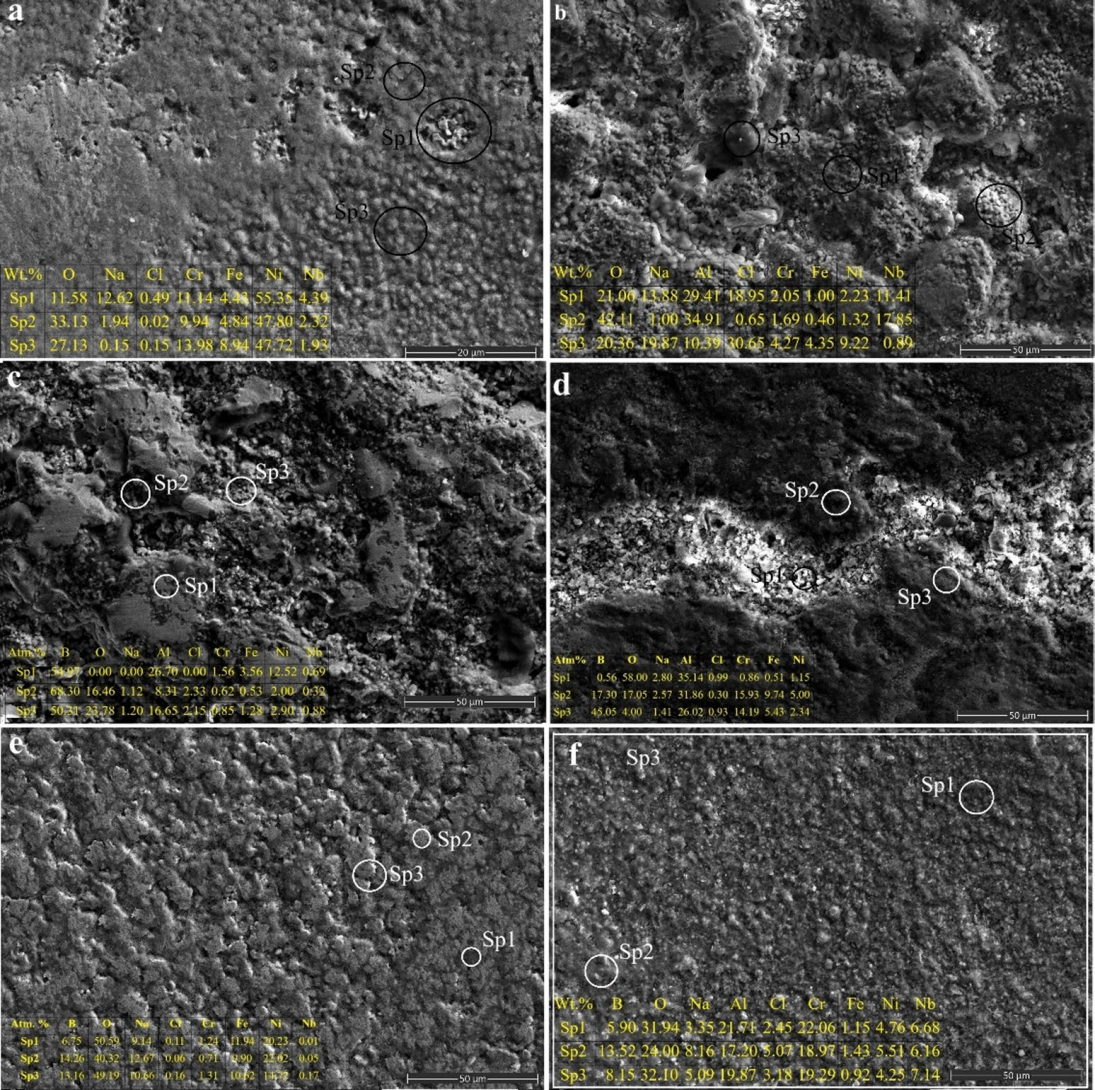
Surface SEM views of samples after corrosion testing and EDS analysis taken from some areas **a** As-built Inc718, **b** SSA, **c** SAB, **d** CBA, **e** SSB, **f** SBA.

Considering Fig. 9a the corroded as-built surface displays shallow pits and interconnected cavities mainly originating from SLM-related unmelted particles and melt-pool irregularities^8,11,48,56^. These defects allow chloride ions to penetrate easily, weakening the naturally formed passive oxide film^8,11,48^. EDS shows high Ni–Cr–Fe matrix composition with noticeable Na and Cl accumulation, confirming active chloride adsorption^11,48,57^. In line with its moderate corrosion rate (1.86 × 10⁻² mm/y), the naturally formed oxide film provides limited protection^11,57^. Although the as-built state displayed the most positive OCP (–0.23 V), the relatively high Icorr (7.80 × 10⁻⁴ A/cm²) indicates insufficient passivity. Thus, it can be said that corrosion initiates preferentially at structural defects, where electrolyte becomes trapped, promoting localized anodic dissolution in a pit-dominated mechanism^8,11,48,56,57^.

The SSA surface (Fig. 9b) shows cracked and fragmented oxide islands, indicating partial breakdown of the Al₂O₃-rich coating under chloride attack. EDS confirms 10–34 at% Al enrichment but also elevated Na and Cl content, revealing electrolyte penetration into microcracks. OCP values shifted to − 0.39 to − 0.47 V, consistent with unstable passivation. However, its corrosion rate (9.1 × 10⁻² mm/y) remains lower than the untreated alloy due to reduced anodic kinetics provided by the alumina layer. The discontinuous nature of the aluminide film allows crevice-like corrosion to occur locally^58–60^.

SAB exhibits significant surface degradation with extensive porosity and particle detachment (Fig. 9c). EDS shows highly heterogeneous B–Al distribution (B reaching > 50 at% locally) and pronounced Na–Cl accumulation, confirming severe electrolyte entrapment. This morphology aligns with its extremely negative OCP (–0.48 V) and the highest corrosion current density (6.15 × 10⁻² A/cm²), resulting in an extreme corrosion rate of 1.46 mm/y. The multilayer, poorly integrated aluminide–boride structure generates abundant micro-galvanic couples and loss of passivity^53^. Corrosion propagates rapidly through deep pores, making SAB the least corrosion-resistant coating both thermodynamically and kinetically.

CBA shows the most compact and uniform post-corrosion surface among all coatings (Fig. 9d). SEM images reveal a dense microstructure with minimal cracking, and EDS indicates homogeneous B–Al–O enrichment combined with very low Na and Cl incorporation (< 1 at%). Its OCP (≈–0.36 V) remains relatively noble and stable, while the corrosion current density is among the lowest (5.65 × 10⁻⁴ A/cm²), yielding a small corrosion rate (1.35 × 10⁻² mm/y). The co-deposited layer forms an adherent, chemically integrated barrier that effectively suppresses pit initiation^32^. Corrosion mainly remains superficial due to the limited electrolyte penetration, confirming CBA as one of the top-performing coatings.

SSB displays a porous boride morphology that partially facilitates electrolyte infiltration (Fig. 9e). EDS indicates high B content (≈ 30–55 at%), yet Na and Cl enrichment remain moderate, consistent with its intermediate corrosion resistance. OCP values around − 0.30 V suggest moderate thermodynamic stability, while Tafel data indicate a corrosion rate of 8.87 × 10⁻² mm/y. Although boride phases such as Ni₂B and CrB offer chemical stability, their intrinsic porosity enables localized chloride accumulation and sub-surface attack^46,47,49^. Corrosion advances mainly through pore-driven pathways, making SSB superior to SAB but less protective than aluminide-containing coatings (SSA, CBA, SBA).

SBA presents a relatively smooth but locally pitted surface (Fig. 9f), reflecting synergistic protection from the boride–aluminide coatings^32,55^. EDS shows enriched Al (≈ 25–40 at%) with moderate B levels and substantially lower Na–Cl incorporation compared with SAB and SSA. Although its OCP is fairly negative (≈–0.47 V), Tafel results reveal the lowest corrosion current density (1.73 × 10⁻⁶ A/cm²) and high Rp (3.06 × 10⁴ Ω), indicating excellent kinetic resistance. The dense Al₂O₃ outer layer adheres strongly to the mechanically robust boride sublayer, limiting electrolyte migration^32,55^. Corrosion proceeds only minimally, making SBA one of the best-performing treatments overall.

## Discussions

The superalloy Inconel 718, produced by Selective Laser Melting (SLM), offers significant advantages such as the fabrication of complex geometries, high material efficiency, rapid prototyping, and fine dendritic microstructures with improved mechanical properties^8,11,13^. Very high cooling rates (~ 10⁶ K/s) promote the homogenization of the γ-matrix, while fine-columnar grains improve fatigue and high-temperature properties^40,43^. However, process-induced heterogeneities such as micro-segregation, Laves phase formation, incompletely melted particles, and microporosity weaken the passive layer continuity in chloride-containing media and thus limit the corrosion behavior^11,14,48^. The depressions and particle pores observed in the as-built sample (Fig. 1a) correspond to the typical behavior of SLM-IN718 and increase its susceptibility to pitting corrosion^40,43^. Thus, the design freedom offered by SLM necessitates targeted surface modification for corrosion protection^8,14^.

Various post-processing strategies have been employed to improve the SLM microstructure, including STA treatments, stress-relief annealing, HIP, electrochemical polishing, and laser remelting^8,14–17^. While HIP-based processes reduce porosity and promote passive film development^8^, strong delta phase formation in HT-STA can trigger local galvanic effects and impair pitting resistance^8,10^. Although electropolishing reduces surface roughness, it offers only limited long-term protection^14,48^. Since these processes do not create a chemically novel, highly stable barrier, thermochemical diffusion coatings are considered a more efficient solution.

Boronizing, aluminizing, and boroaluminizing have been successfully employed to improve the corrosion, oxidation, and wear resistance of nickel-based superalloys. Although the process was carried out at at high temperature (900–1000 °C), the reaction was limited to the surface region, and boride and/or aluminide diffusion layers, along with an interdiffusion zone, were formed. The SLM-induced γ-matrix dendritic microstructure, formed due to the SLM process, was present at the core, indicating that the surface microstructural features were altered due to the thermochemical surface treatments. SEM-EDS and XRD studies (Figs. 2, 3, 4 and 5, and 6) revealed the characteristic phase formation due to each process. SSA process leads to the formation of NiAl/Ni₃Al-Al₂O₃ layers, an SSB process leads to the formation of multilayer borides, a CBA process leads to the formation of compact boron aluminide layers, an SAB process leads to the formation of a heterogeneous and porous phase ensemble, and an SBA process leads to the formation of an aluminide-rich top layer over a boride base layer. Among these layers, the highest continuity and homogeneity were found in the CBA and SBA layers, which is important for corrosion resistance, as revealed by Tang et al. (2024).

SSA produces NiAl/Ni₃Al-Al₂O₃ layers; SSB leads to multilayer borides; CBA yields compact boroaluminide structures; SAB results in a heterogeneous, partially porous phase ensemble; SBA forms an aluminide-enriched top layer over a boride layer. The highest layer continuity and elemental homogeneity were observed, particularly with CBA and SBA crucial for corrosion resistance and consistent with Tang et al. (2024). The higher grain boundary density of SLM-IN718 promotes faster diffusion and thus more pronounced layer formation compared to rolled or cast IN718^25–32,61^. Electrochemically, SSB exhibits only moderate performance due to some porosity, while SSA, although passivated, does not achieve complete coverage. CBA and especially SBA achieve significantly better corrosion parameters than many coatings on conventional IN718 substrates^32^; the very low Icorr value of SBA (1.73 × 10⁻⁶ A/cm²) underscores the excellent electrochemical stability of the SLM-coated systems.

The OCP and Tafel analyses clearly demonstrate that layer continuity and porosity are the key factors influencing corrosion behavior. While SSA and SSB exhibit moderate protective effects and SAB performs worst, CBA and especially SBA achieve the highest thermodynamic and kinetic stability (OCP, Icorr, Rp) due to synergistic boroaluminide phases. The achieved values ​​of Rp = 3.06 × 10⁴ Ω and Icorr = 1.73 × 10⁻⁶ A/cm² point to a promising surface engineering concept for applications in marine, energy, chemical process engineering, and additively manufactured turbine components. The main achievement of this work lies in the systematic evaluation of five thermochemical coating routes on the same SLM IN718 substrate and the demonstration that boroaluminide coatings exploit the microstructural advantages typical of SLM to achieve superior corrosion resistance.

## Conclusions

This study systematically compared aluminizing (SSA), boronizing (SSB), co-deposited boroaluminizing (CBA), and sequential SAB/SBA diffusion coatings on SLM-Inconel 718. Microstructural, phase and electrochemical findings clearly demonstrate the decisive role of coating architecture on chloride-induced corrosion. The main conclusions are as follows:

- XRD/SEM–EDS analyses showed that SSA formed NiAl/Ni₃Al/FeAl₂ with an Al₂O₃ scale, while SSB produced multilayer Ni₂B–FeB–CrB borides. CBA generated dense aluminoboride phases (Ni₂₀Al₃B₆). Sequential routes diverged sharply: SAB yielded a porous, heterogeneous mix, whereas SBA produced a boride base with a continuous aluminide/Al₂O₃ top layer. CBA and SBA exhibited the highest structural continuity.
- Corrosion resistance ranked as: SBA < CBA < SSA < SSB < as-built < SAB. SBA achieved the best performance (Icorr = 1.73 × 10⁻⁶ A/cm²; Rp = 3.06 × 10⁴ Ω). CBA also showed low current density and noble Ecorr. SAB was the worst due to extreme heterogeneity and microgalvanic acceleration. SSA and SSB offered only moderate improvement because of partial film continuity.
- Post-corrosion SEM revealed fragmented Al₂O₃ in SSA, electrolyte-penetrating porosity in SSB, and severe crack-driven dissolution in SAB. CBA preserved the most compact surface, while SBA showed minimal pit formation due to its mechanically robust boride layer and chemically stable Al₂O₃ scale.
- As-built IN718 failed by pit initiation at SLM defects. SSA degraded via cracked Al₂O₃ islands; SSB via porosity-driven crevice attack; SAB through interconnected dissolution pathways. CBA minimized pit propagation through its compact aluminoboride matrix, while SBA suppressed attack through synergistic mechanical (boride) and chemical (Al₂O₃) stability.
- SBA and CBA coatings provide the most reliable protection for SLM-Inconel 718 in chloride media. Their superiority stems from dense Al–B diffusion layers, stable Al₂O₃ scales, high mechanical integrity, and minimized porosity. These hybrid aluminide–boride systems effectively mitigate the intrinsic microstructural weaknesses of SLM parts, making them strong candidates for aerospace, turbine, and marine components requiring durable corrosion resistance.

## Data Availability

The data that support the findings of this study are available from the corresponding author upon reasonable request.
